# The Phenotypic Plasticity of Duplicated Genes in *Saccharomyces cerevisiae* and the Origin of Adaptations

**DOI:** 10.1534/g3.116.035329

**Published:** 2016-10-31

**Authors:** Florian Mattenberger, Beatriz Sabater-Muñoz, Christina Toft, Mario A. Fares

**Affiliations:** * Department of Abiotic Stress, Instituto de Biología Molecular y Celular de Plantas, Consejo Superior de Investigaciones Científicas, Universidad Politécnica de Valencia, 46022 Spain; † Department of Genetics, Smurfit Institute of Genetics, University of Dublin, Trinity College, Dublin 2, Ireland; ‡ Department of Genetics, University of Valencia, 46100 Burjassot, Spain; § Departamento de Biotecnología, Instituto de Agroquímica y Tecnología de los Alimentos, Consejo Superior de Investigaciones Científicas, Valencia, 46980 Paterna, Spain

**Keywords:** evolutionary biology, gene function, small-scale duplicates, whole-genome duplicates, transcriptional profiles

## Abstract

Gene and genome duplication are the major sources of biological innovations in plants and animals. Functional and transcriptional divergence between the copies after gene duplication has been considered the main driver of innovations . However, here we show that increased phenotypic plasticity after duplication plays a more major role than thought before in the origin of adaptations. We perform an exhaustive analysis of the transcriptional alterations of duplicated genes in the unicellular eukaryote *Saccharomyces cerevisiae* when challenged with five different environmental stresses. Analysis of the transcriptomes of yeast shows that gene duplication increases the transcriptional response to environmental changes, with duplicated genes exhibiting signatures of adaptive transcriptional patterns in response to stress. The mechanism of duplication matters, with whole-genome duplicates being more transcriptionally altered than small-scale duplicates. The predominant transcriptional pattern follows the classic theory of evolution by gene duplication; with one gene copy remaining unaltered under stress, while its sister copy presents large transcriptional plasticity and a prominent role in adaptation. Moreover, we find additional transcriptional profiles that are suggestive of neo- and subfunctionalization of duplicate gene copies. These patterns are strongly correlated with the functional dependencies and sequence divergence profiles of gene copies. We show that, unlike singletons, duplicates respond more specifically to stress, supporting the role of natural selection in the transcriptional plasticity of duplicates. Our results reveal the underlying transcriptional complexity of duplicated genes and its role in the origin of adaptations.

Gene duplication has been a major driving force of biological innovation in plants (Cui *et al.* 2006; Carretero-Paulet and Fares 2012; Holub 2001; Lespinet *et al.* 2002; Otto and Whitton 2000; Wendel 2000; Kim *et al.* 2004) and animals (Otto and Whitton 2000; Hoegg *et al.* 2004). Arguably, understanding how gene duplication gives origin to novel functions and adaptations is a fundamental aim of evolutionary biology. The functional and transcriptional divergence between the gene copies of a duplicated gene has been proposed to facilitate the origin of novel functions (Conant and Wolfe 2008; Lynch and Conery 2000; Ohno 1999, 1970). However, the tempo and mode of each divergence kind and the interplay between both remains largely unexplored.

Ohno proposed that after the duplication of a gene, the emerging genetic redundancy leads to relaxed selection against one of the gene copies while the other copy remains under strong purifying selection (Ohno 1970, 1999). The selectively relaxed gene copy explores novel genotypes, many of which will be deleterious and lead to the loss of the rapidly evolving gene copy (Lynch and Conery 2003). A less likely scenario is the preservation of both copies by purifying selection after a period of relaxed selection leading to novel functions in the form of sub- or neo-functionalization (Ohno 1970, 1999; Lynch and Conery 2003; Taylor and Raes 2004). Particular scenarios for this general model of the functional divergence of gene copies have been proposed (Des Marais and Rausher 2008; Force *et al.* 1999; Innan and Kondrashov 2010). Classic theory has also given credit to the expression divergence between gene copies as a prerequisite for the preservation of genes in duplicate and the eventual finding of new functions (Ferris and Whitt 1979; Force *et al.* 1999; Ohno 1970). Moreover, previous studies have found a genome-wide transcriptional response of *Saccharomyces cerevisiae* to a wide range of environmental perturbations (Ferea *et al.* 1999; Causton *et al.* 2001; Cormier *et al.* 2010; Ideker *et al.* 2001; Landry *et al.* 2006; Stern *et al.* 2007).

The rapid evolution of gene expression after duplication (Li *et al.* 2005; Thompson *et al.* 2013) suggests an adaptive role for the transcriptional plasticity of duplicates. However, the question remains open whether duplicates follow the general response patterns to stresses that are shown by singleton genes or, alternatively, they have allowed the origin of stress-specific adaptations that have been favored by natural selection. It also remains obscure whether the transcriptional plasticity of duplicates has driven their functional specialization. Understanding this plasticity through studies like the one conducted here provides a much wider picture of the role of gene duplication in the origin of adaptations and ecological diversification.

Gene duplication in plants has been followed by rapid expression divergence between gene copies (Blanc and Wolfe 2004; Ha *et al.* 2007, 2009; Wang *et al.* 2012). Since most duplicated genes are thought to mediate the interaction between the organism and environment, their expression changes have been suggested to be strongly linked to generating adaptations rather than responding to developmental perturbations (Ha *et al.* 2007). Most importantly, expression divergence has been seen to correlate with the sequence divergence between duplicate gene copies in plants (Blanc and Wolfe 2004) and, although less clearly (Wagner 2000a), in yeast (Gu *et al.* 2002). Two questions remain unexplored: (a) are duplicated genes more transcriptionally plastic than anticipated?; and (b) does transcriptional plasticity determine the functional fates of gene copies? Answering these questions would reveal the potential of gene duplicates to expedite adaptations.

The Baker’s yeast *S. cerevisiae* duplicated its genome roughly 100 MYA (Wolfe and Shields 1997) triggered by the possible hybridization between different yeast species (Marcet-Houben and Gabaldon 2015; Wolfe 2015). Only 1120 pairs of duplicates have been retained, of which 554 belong to the whole-genome duplication event and the remaining are classified as duplications of small scale (Fares *et al.* 2013). Many of the yeast-duplicated genes enable the growth of *S. cerevisiae* under stressful conditions, the genetic basis of which has enabled the exploitation of the biotechnological benefits of yeast in the multimillionaire wine industry. The genetic and biotechnological properties of this yeast offer a unique opportunity to study the role of gene duplication in innovation. In this study, we explore whether the transcriptional plasticity of duplicated genes in *S. cerevisiae* has contributed to the origin of adaptations to stress and functional specialization of duplicate gene copies. We address this question by exhaustively and extensively analyzing the expression pattern dynamics of duplicated genes in the yeast *S. cerevisiae* after subjecting it to a number of stress conditions. Here, we find that not only duplicates are more transcriptionally polymorphic as concluded before (Ha *et al.* 2009) but that they are more transcriptionally plastic than singletons under environmental stress. This transcriptional plasticity increases after gene duplication and it is strongly correlated with the functional divergence of duplicate gene copies. The study of the patterns of sequence divergence, functional interactions, and transcriptional plasticity of duplicates makes possible the identification of stress-specific as well as general transcriptional response patterns. We show that, unlike singleton genes, duplicates have given origin to stress-specific adaptations. Our data describe a complex dynamic of transcriptional evolution following the gene and genome duplications of a simple eukaryotic organism and reveal the origins of yeast adaptations.

## Materials and Methods

### Identification of duplicated genes

Paralog pairs of duplicated genes were identified as the resulting best reciprocal hits from all-against-all BLAST searches using BLASTP with an E-value cutoff of 1E-5 and a 50 bit score (Altschul *et al.* 1997). Paralogs were then divided into two groups according to the mechanism of their origin: whole-genome duplications (WGDs) and small-scale duplications (SSDs). WGDs are those extracted from the reconciled list provided by the Yeast Gene Order Browser (YGOB, http://wolfe.gen.tcd.ie//ygob; Byrne and Wolfe 2005) (555 pairs of genes), and these were not subjected to subsequent SSD. All other paralogs were considered to belong to the category of SSDs (560 pairs of genes). The duplicates used in this study have been estimated to have their origin on the time point of the WGD that took place 100 MYA (Wolfe and Shields 1997). Also, in this study we have used the SSDs that exhibit similar distribution of synonymous substitutions as those of WGDs, so roughly belonging to the same age (Fares *et al.* 2013; Keane *et al.* 2014).

### Sequence alignments and analysis of divergence

For each protein-coding gene of *S. cerevisiae* we searched for its ortholog in the closely related species *S. paradoxus* using the program blastP. Pairwise sequence alignments were built using the program ClustalW. To calculate the distance between *S. cerevisiae* and *S. paradoxus* for each of the genes, we estimated the number of nonsynonymous nucleotide substitutions per nonsynonymous site (d_N_), synonymous substitutions per synonymous site (d_S_), and the nonsynonymous-to-synonymous rates ratio (ω = d_N_/d_S_) using the maximum-likelihood approach under the Goldman and Yang model (Goldman and Yang 1994) as implemented in the PAML package version 4.7 (Yang 2007).

### Analysis of gene expression in S. cerevisiae

The transcriptomic profiling was performed in the *S. cerevisiae* Y06240 haploid *msh2* deletion strain (BY4741; *Mata*; *his3**D1*; *leud2**DO*; *met15**DO*; *ura3**DO*; *msh2*::*kanMX4*) (Fares *et al.* 2013), with three technical replicates for each biological stress condition [3% lactic acid (YPL), 3% ethanol (YPE), 3% glycerol (YPG), 0.25 mM H_2_O_2_ (YPOx), 0.25 mM H_2_O_2_ + 1.5% dextrose (YPOxD)] in comparison with the normal growth condition (Yeast extract, Peptone, Dextrose media). Total RNA extractions were performed with RNeasy kit (Qiagen) following manufacturer instructions. Ribosomal RNA was removed using the Ribo-Zero Gold rRNA removal yeast (Illumina) depletion kit. Stranded RNA libraries were constructed using TruSeq stranded mRNA (Illumina) from oligo-dT captured mRNAs from depleted samples. Libraries were run in NextSequation 500 (Illumina) at 75 nt single read using High Output 75 cycles kit v2.0 (Illumina).

The treatment of the RNA libraries was done following a previous study in which different methods of differential expression analyses were compared (Zhang *et al.* 2014). RNA libraries were sequenced at the Genomic core facility at Servicio Central de Soporte a la Investigación Experimental (SCSIE), University of Valencia, Spain. Raw reads were analyzed using FastQC report and cleaned with CutAdapt as implemented in RobiNA software package v 1.2.4 (Lohse *et al.* 2012). Low-quality reads were filtered and trimmed (Phred score <20 and size <40 nt were discarded). Since we had a reference transcriptome from S288c strain, reads were then aligned with Bowtie (up to two mismatches accepted) to the reference transcriptome (PRJNA290217) from the reference S288c strain. Statistical assessment of differential gene expression was done either with edgeR (Robinson *et al.* 2010) or with DESeq (Anders and Huber 2010) as implemented in RobiNA. A previous study compared the different expression analysis methods, concluding that edgeR and DESeq were the best-performing methods when the objective is to analyze differential expression (Zhang *et al.* 2014). Comparison of logarithmic fold-change of our expression data between edgeR and DESeq provided very strong correlation (Spearman correlation coefficient: *ρ* = 0.995, *P* < 2.2 × 10^−16^, Figure 1 of Supplemental Material, File S8). Significant expression changes were identified using a false discovery rate (FDR <0.05). These results indicate that our quantification of expression data is robust to the method used. All newly sequenced RNA sequences are available from the Sequence Read Archive with the following accession number: SRP074821.

### Genetic interaction data

We used the latest update of the genetic functional chart of *S. cerevisiae* (Costanzo *et al.* 2010) (File S4 and File S5 from http://drygin.ccbr.utoronto.ca/∼costanzo/). The genetic map is based on the synthetic genetic array methodology (Tong *et al.* 2001). In this methodology, synthetic lethal genetic interactions are systematically mapped to single and double mutants. In this study, two genes are considered to interact genetically if the double knockout mutant of the two genes has significantly larger or smaller effect than the multiplicative effects of simple knockouts.

### Software

Calculations and statistics were performed using MS Excel and R 3.2.1, unless otherwise indicated. Data management was possible using in-house built Perl scripts.

### Data availability

Strains are available upon request. All RNA sequences are available from the Sequence Read Archive (accession number SRP074821). File S1 contains the significant transcriptionally altered genes in *S. cerevisiae* upon growing in ethanol stress. File S2 contains the significant transcriptionally altered genes in *S. cerevisiae* upon growing in glycerol stress. File S3 contains the significant transcriptionally altered genes in *S. cerevisiae* upon growing in acidic stress. File S4 contains the significant transcriptionally altered genes in *S. cerevisiae* upon growing in oxidative stress. File S5 contains the significant transcriptionally altered genes in *S. cerevisiae* upon growing in oxidative stress in a growth medium supplemented with dextrose. File S6 contains the list of duplicated genes in *Candida glabrata*. File S7 contains the conservation indices of promoter alignments for duplicated genes with altered transcriptional profiles. File S8 compares the methods edgeR and DESeq for the calculation of reads mapped to each gene.

## Results

To test the role of duplicated genes of *S. cerevisiae* in the origin of adaptations, we sequenced the transcriptome of a haploid *msh*2 deletion strain after growing it in normal YPD medium and under five different stress conditions: (a) ethanol, (b) glycerol, (c) lactate, (d) oxidative stress, and (e) oxidative stress in a medium supplemented with dextrose (see *Materials and Methods*). Subsequently, we compared the transcriptional modifications of *S. cerevisiae msh2*::*kanMX4* under each of the conditions and sought to investigate the role of duplicated genes in displaying transcriptional plasticity under stress. We used this strain because it has been allowed to evolve for hundreds of generations in YPD medium, hence is adapted to this medium, and allows the maintenance of population genetic polymorphism due to its higher mutation rate compared to the wild-type strain.

### Duplicated genes exhibit significant transcriptional plasticity under stress

After growing biological replicates of the yeast populations under normal and each of the five different stress conditions, we extracted total RNA for RNAseq library construction and identified differentially expressed (DE) genes based on the comparison of their expression levels relative to normal conditions (see *Materials and Methods*). In total, we obtained reliable RNA sequence data for 5825 genes in the YPD medium (normal conditions) and each of the five stress conditions (File S1, File S2, File S3, File S4, and File S5), of which an important fraction was significantly altered under stress conditions (Table 1).

**Table 1:** Transcription alterations under stress conditions

Stress	Comparison	Number of Genes of First Type (%)	Number of Genes of Second Type (%)	Odds Ratio (*F*)	Probability
Ethanol	D*^a^* *vs.* S*^b^*	907 (40.5%)	1341 (29.3%)	1.64	<2.2 × 10^−16^
	WGDs*^c^* *vs.* S	515 (47.6%)	1341 (29.3%)	2.12	<2.2 × 10^−16^
	SSDs*^d^* *vs.* S	392 (33.9%)	1341 (29.3%)	1.27	8.1 × 10^−4^
	WGDs *vs.* SSDs	515 (47.6%)	392 (33.9%)	1.68	2.7 × 10^−9^
Glycerol	D *vs.* S	1134 (50.6%)	1693 (36.9%)	1.75	<2.2 × 10^−16^
	WGDs *vs.* S	617 (56.9%)	1693 (36.9%)	2.18	<2.2 × 10^−16^
	SSDs *vs.* S	517 (44.7%)	1693 (36.9%)	1.41	2.3 × 10^−7^
	WGDs *vs.* SSDs	617 (56.9%)	517 (44.7%)	1.10	0.21
Lactate	D *vs.* S	1038 (46.3%)	1471 (32.1%)	1.83	<2.2 × 10^−16^
	WGDs *vs.* S	571 (52.7%)	1471 (32.1%)	2.28	<2.2 × 10^−16^
	SSDs *vs.* S	467 (40.4%)	1471 (32.1%)	1.47	2.6 × 10^−8^
	WGDs *vs.* SSDs	571 (52.7%)	467 (40.4%)	1.56	2.3 × 10^−7^
Oxidative	D *vs.* S	42 (1.9%)	59 (1.3%)	1.46	0.06
	WGDs *vs.* S	29 (2.7%)	59 (1.3%)	2.07	0.002
	SSDs *vs.* S	12 (1.1%)	59 (1.3%)	0.82	0.65
	WGDs *vs.* SSDs	29 (2.7%)	12 (1.1%)	2.54	0.007
Oxidative + dextrose	D *vs.* S	1064 (47.5%)	1574 (34.4%)	1.73	<2.2 × 10^−16^
	WGDs *vs.* S	589 (54.4%)	1574 (34.4%)	2.20	<2.2 × 10^−16^
	SSDs *vs.* S	475 (41.1%)	1574 (34.4%)	1.36	5.1 × 10^−6^
	WGDs *vs.* SSDs	589 (54.4%)	475 (41.1%)	1.61	2.2 × 10^−8^

a Duplicated genes.

b Singleton genes.

c Whole-genome duplicates.

d Small-scale duplicates.

**Figure 1:**
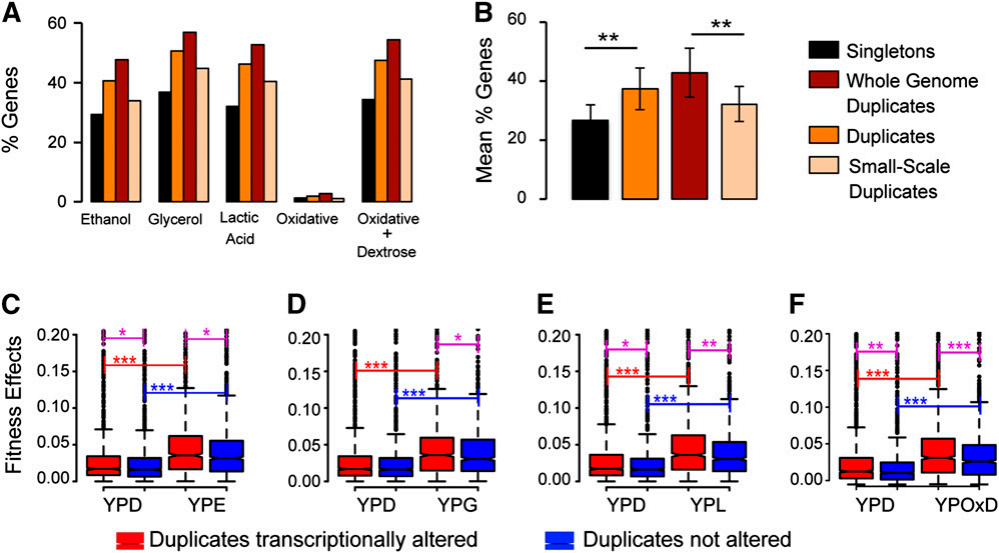
Duplicated genes exhibit higher transcriptional plasticity than singleton genes and are involved in adaptation. (A) Percentage of genes with transcriptional flexibility when *S. cerevisiae* is grown under each of the five stress conditions tested in this study: ethanol stress, glycerol stress, acidic stress by lactate, oxidative stress, and oxidative stress in a medium supplemented with dextrose. (B) The mean percentage of genes of the categories’ singletons (black bar), duplicates (orange bar), duplicates generated by whole genome duplication (red bar), and duplicates generated by small-scale duplication (light yellow bar) with transcriptional alterations in the five stress conditions tested in this study. (C–F) We measured the contribution of transcriptionally altered duplicates to the fitness of *S. cerevisiae* under YPD and stress growth conditions using knock down gene data from Steinmetz *et al.* (2002). We then compared the fitness contribution of these altered duplicates (red boxes) with that of duplicates with no evidence for transcriptional plasticity under stress (blue boxes). These comparisons were performed for the sets of altered and not-altered duplicates identified under ethanol stress (C), glycerol stress (D), lactate stress (E), and oxidative stress supplemented with dextrose (F). Significant differences are indicated with *, **, and *** when the difference was significant at the levels of 0.01, 0.001, and 0.0001, respectively, using a Mann–Whitney *U*-test.

In all stress conditions, there was a significant transcriptomic response affecting 2248, 2827, 2509, 101, and 2638 genes under stress induced by ethanol, glycerol, lactate, oxidative stress, and oxidative stress in a medium supplemented with dextrose, respectively (File S1, File S2, File S3, File S4, and File S5). The response affected duplicates significantly more than singletons in all stress conditions, although the low number of altered genes limited the power of the test in the case of oxidative stress (Table 1 and Figure 1A). The mechanism of duplication, including WGD and SSD, also made a difference, with WGDs being more significantly enriched for altered expression genes than SSDs (Table 1 and Figure 1B). Taking all nonredundant transcriptomic responses together for all stress conditions, duplicated genes showed significantly larger increments of transcription under stress than singleton genes (Figure 1B). Indeed, on average 837 duplicated genes out of the 2240 duplicates (37.4%) in *S. cerevisiae* exhibited significant increments of expression against 1227 out of 4580 singletons (26.8%) (Fisher’s exact test: odds ratio *F* = 1.63, *P* < 2.2 × 10^−16^). We identified an average of 42.2%, over all the stresses of 464 duplicates out of 1100 WGDs, to be transcriptionally plastic, a proportion significantly higher than that for singletons (Fisher’s exact test: odds ratio *F* = 1.99, *P* < 2.2 × 10^−16^). Likewise, the proportion of transcriptionally plastic SSDs (an average of 372 out of 1140 SSDs, 32.6%) was significantly higher than that for singletons (Fisher’s exact test: odds ratio *F* = 1.32, *P* = 1.1 × 10^−4^). WGDs also presented a significantly higher proportion of transcriptionally plastic genes than SSDs (Fisher’s exact test: odds ratio *F* = 1.51, *P* = 3.5 × 10^−6^).

An alternative explanation for the adaptive value of the transcriptional plasticity of duplicates under stress is a low contribution of duplicates to fitness, with their response being the reflection of transcriptional noise caused by environmental perturbations. To test this possibility, we calculated the contribution of each duplicated gene to the fitness of *S. cerevisiae* under normal and stress conditions taking previously published fitness data (Steinmetz *et al.* 2002). To this end, we subtracted the normalized fitness values of a strain after a gene was deleted or knocked down under stress from the fitness of its ancestral strain (*i.e.*, 1). Therefore, large fitness absolute increment values indicate that the contribution of the gene to fitness is high under those conditions.

We first compared the contribution to fitness of duplicates with and without altered transcriptomic profiles in YPD. Transcriptionally altered duplicates exhibited higher contribution to fitness than unaltered duplicates (Figure 1, C–F), discarding the possibility that altered duplicates may have less contribution to fitness than not-altered duplicates consequently being less selectively constrained to change. Both transcriptionally altered and not-altered duplicates showed a significant increase in their contribution to fitness under stress (Figure 1, C–F). However, this increase was sharper in transcriptionally altered duplicates than in unaltered duplicates.

### Increased transcriptional plasticity after gene duplication

The higher transcriptional plasticity of duplicated genes in *S. cerevisiae* when compared to singletons may be the result of a biased preservation in duplicate of highly transcriptionally plastic genes. To test whether gene duplication increases transcriptional plasticity we examined the patterns of transcriptional plasticity of duplicates and singletons in the post-WGD yeast *C. glabrata*, a phylogenetically close species to *S. cerevisiae*. To this end, we asked the question of whether duplicates of *S. cerevisiae* had singleton orthologs in *C. glabrata* that were not more transcriptionally plastic than expected when compared to other *C. glabrata* singletons under stress and vice versa. We obtained RNA sequence data from a previous publication in which transcriptomic data were available under YPD conditions and under acidic stress (Linde *et al.* 2015), similar to our data on lactic acid stress. *S. cerevisiae* orthologs from *C. glabrata* were identified using syntheny information available in the pillars of YGOB (Byrne and Wolfe 2005). In total, we identified 4844 reliable *S. cerevisiae*: *C. glabrata* orthologs. Of these 4844 orthologs, 788 genes in *C. glabrata* were duplicated genes (394 pairs, File S6), of which 123 were duplicated in *C. glabrata* but not in *S. cerevisiae*. Of the 2240 duplicates of *S. cerevisiae*, we found 1019 orthologs that were singletons in *C. glabrata*. We first asked whether singletons in *C. glabrata* that are orthologs of duplicates in *S. cerevisiae* exhibit higher transcriptional plasticity than singletons in *C. glabrata* with no duplicate orthologs in *S. cerevisiae*. If this were the case, then gene duplication would have no role in transcriptional plasticity in *S. cerevisiae*. Notwithstanding that the transcriptional plasticity for a particular gene may vary among species, we found that the percentage of singletons with significant transcriptional alterations under stress in *C. glabrata* that are orthologs to *S. cerevisiae* duplicates (599 out of a total of 1019 genes, 58.7%) was not significantly higher than that of transcriptionally altered singletons in *C. glabrata* that had no duplicate orthologs in *S. cerevisiae* (1725 out of a total of 3062 singleton genes, 56.3%) (Fisher exact test: odd’s ratio *F* = 1.10, *P* = 0.17). Conversely, duplicates in *C. glabrata* that were orthologs to singletons in *S. cerevisiae* exhibited a significantly higher percentage of transcriptionally altered genes under stress (82 out of a total of 123 genes, 66.7%) than singletons in *C. glabrata* (Fisher’s exact test: odd’s ratio *F* = 1.55, *P* = 0.02).

### Differential patterns of transcriptional alterations within duplicated genes

**Figure 2:**
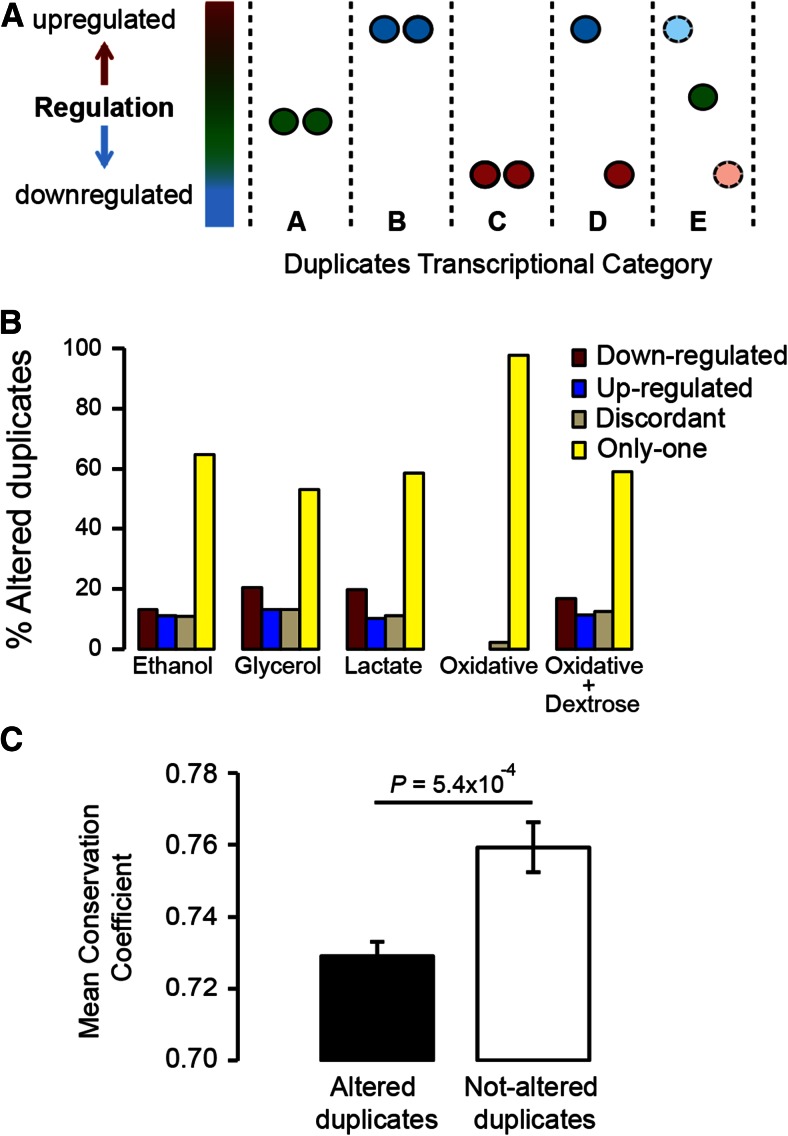
Duplicated genes exhibit differential patterns of transcriptional plasticity under stress. We identified five patterns of transcriptional plasticity for the duplicates of *S. cerevisiae* growing under stress conditions (A), including duplicates in which neither copy has been altered (category A), those with both copies up-regulated (category B), those with both copies down-regulated (category C), those with gene copies showing discordant transcriptional plasticities (category D), and those in which only one gene copy is altered while its sister copy is unaltered under stress (category E). Calculating the percentage of the duplicated genes belonging to each of the transcriptional categories (B), we found that the category with only one copy altered (yellow bar) is the one showing the highest percentage of the altered duplicates under stress. (C) We measured the conservation of the promoter regions for altered and not-altered duplicates and found that altered duplicates (black bar) exhibit lower conservation than not-altered duplicates under stress (white bar).

We sought to investigate the different transcriptional profiles of pairs of duplicated genes and their contributions to the fitness of *S. cerevisiae*. We divided duplicated genes that underwent transcriptional alterations after stress into five different categories (Figure 2A and Table 2): (a) duplicates in which both of the gene copies were up-regulated under stress (called herein Up pattern); (b) duplicates with both copies down-regulated under stress (Down pattern); (c) duplicates with one copy up-regulated and one copy down-regulated under stress (Discordant pattern), (d) duplicates with one copy showing not-altered transcription under stress while its sister copy shows either up-regulation or down-regulation under stress (Only-one pattern), and (e) duplicates that remained unchanged under stress (Not-altered pattern).

**Table 2:** Categories of altered expression of duplicates

	Number of Pairs Both Copies Concordant				
Stress	Down	Up	Number of Pairs Discordant	Number of Pairs Not-Altered	Number of Pairs One-Altered
Ethanol	89	76	74	438	677
Glycerol	159	103	102	413	777
Lactic acid	147	76	83	433	739
Oxidative	0	0	1	41	42
Oxidative + dextrose	129	87	96	448	760

In each of the stress conditions, the category “Only-one” comprised the largest number of duplicates with altered transcriptional profiles, with this category including 53–97% of the altered duplicates in the five stresses (Figure 2B). These results support the classical view of evolution by gene duplication, according to which following gene duplication one copy undergoes rapid divergence while the other copy keeps the ancestral function. Here we show that this pattern of evolution by gene duplication is also true for the regulatory evolution of duplicated genes.

Duplicate gene copies with higher transcriptional divergence under stress should show higher sequence divergence when compared to orthologous sequences from other phylogenetically related species if the basis for this transcriptional plasticity was encoded in the gene sequence. To test this hypothesis, we were able to obtain 537 reliable promoter alignments for duplicates in *S. cerevisiae* and at least four additional phylogenetically related yeast species (File S7). The main *Saccharomyces* species we compared *S. cerevisiae* to were *S. bayanus*, *S. castellii*, *S. mikatae*, *S. paradoxus*, *S. kluyveri*, and *S. kudriavzevii*. Not all species presented annotated intergenic regions but we used all those alignments that included at least four of the species. We aligned the 600 nucleotide sequence regions upstream of duplicated genes and their orthologs, as these are likely to include most if not all the regulatory elements of the genes (Ohler and Niemann 2001). We then measured the coefficient of conservation (CC) for each nucleotide site using the entropy equation (Cover and Thomas 2006; Halabi *et al.* 2009; Ruiz-Gonzalez and Fares 2013):

$$CC=f_{k}^{\left(a\right)}\ln\frac{f_{k}^{\left(a\right)}}{q^{\left(a\right)}}+\left(1-f_{k}^{\left(a\right)}\right)\ln\frac{1-f_{k}^{\left(a\right)}}{1-q^{\left(a\right)}};\forall a\in \left[A,T,G,C\right]$$

In this equation, CC of a nucleotide (*a*) at position (*k*) in an alignment is defined as the entropy of the observed frequency of *a* at *k* ($f_{k}^{\left(a\right)}$) relative to the background frequency of *a* in all sequences of the alignment (*q^(a)^*). Therefore, the more conserved the site the higher is its CC value. CC was averaged for each promoter and then these averages were used to compare altered duplicates (those belonging to the categories “Up,” “Down,” “Discordant,” and “Only-one”) with not-altered duplicates (those belonging to the category “Not-altered”). For each of the stress conditions we estimated the CC values for altered and not-altered duplicates. We then pulled all the data together from all stress conditions and compared the CC values of altered to that of not-altered duplicates. The CC values of duplicates with constant transcriptional profiles under stress (mean ± SE = 0.76 ± 0.005) were significantly larger than those of duplicates with altered transcriptional profiles (mean ± SE = 0.72 ± 0.01) (Figure 2C), and the difference was significant using a parametric test (*t*-test: *t* = 3.47, d.f. = 1140.9, *P* = 5.4 × 10^−4^) and a nonparametric test (Mann–Whitney *U*-test: *P* = 0.003), indicating that higher transcriptional plasticity of duplicates may be due to a divergence in their promoter sequences from the ancestral preduplication state.

### Duplicates with different transcriptional divergence patterns exhibit different functional dependencies

To determine whether the transcriptional plasticity of duplicated genes is accompanied by a functional divergence of gene copies, we analyzed the genetic interaction network of *S. cerevisiae* and asked how many of the duplicated genes show genetic interactions between their gene copies, hence are functionally dependent on one another (Costanzo *et al.* 2010), within each of the transcriptional categories (*i.e.*, Up, Down, Discordant, Only one, and Not-altered). To this end, we used the genetic interaction map of *S. cerevisiae* as a proxy to the functions of each of the genes (Costanzo *et al.* 2010). This map contains roughly 6.5 million genetic interactions and the functional chart for 75% of the *S. cerevisiae* genes. The number of genetic interactions for a particular gene is a proxy to the number of functions it performs, as the deletion of both of the genes identified as interacting produces significantly different fitness effects than the multiplicative effect of single gene deletions (Costanzo *et al.* 2010). We identified 762,768 significant genetic interactions (*i.e.*, epistasis, ε) in *S. cerevisiae*, of which 52% were synergistic (*i.e.*, the double mutant exhibited significantly lower fitness W_12_ than the multiplicative effects of individual mutants: ε = W_12_ – W_1_W_2_; ε < 0) and 48% were antagonistic interactions (ε = W_12_ – W_1_W_2_; ε > 0). However, duplicated genes were largely biased regarding the sign epistasis, with the majority of the epistasis (89.5%) being synergistic (binomial test: *P* < 2.2 × 10^−16^). This pattern was also true for transcriptionally altered duplicates (89.74% synergistic epistasis). Dividing transcriptionally altered duplicates into the different categories provides similar results, with all such categories being equally enriched for duplicates with synergistic epistasis: up-regulated duplicates presented largely synergistic epistasis (varying between 86% in ethanol and 93% under oxidative stress supplemented with dextrose), and so did the only-one category (ranging between 87% of the interactions being synergistic under glycerol stress and 92.7% in ethanol stress). These percentages were of the same order in the “Down” and “Discordant” categories.

**Figure 3:**
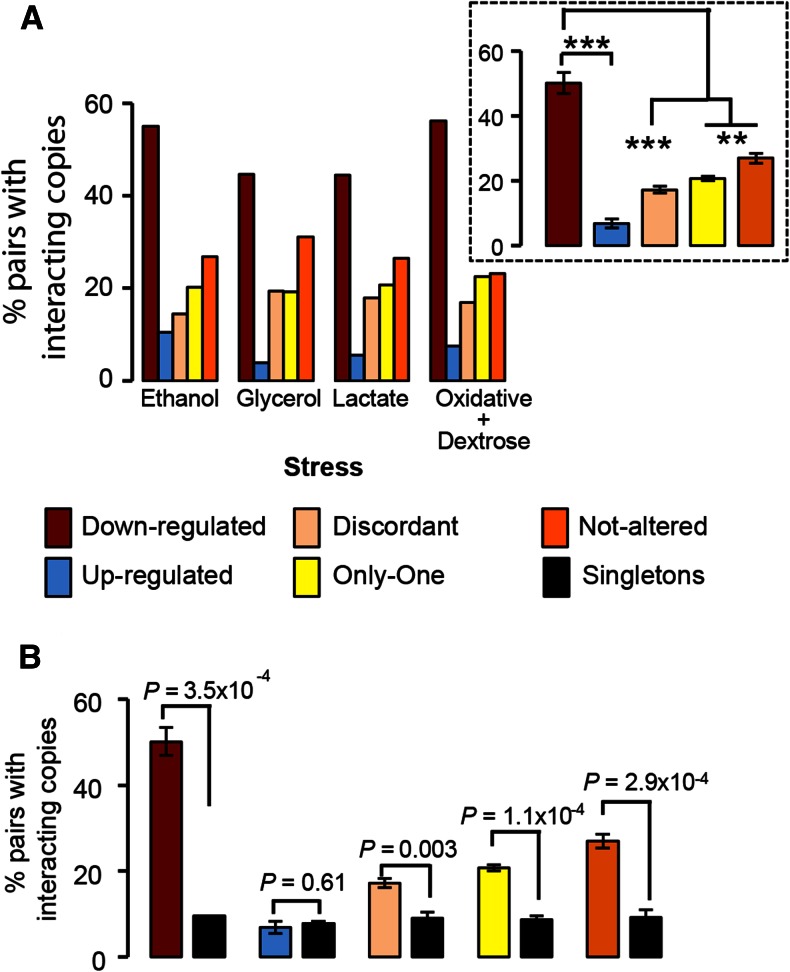
The genetic dependencies between gene copies of transcriptionally plastic duplicates. We measured the number of pairs within each of the transcriptional categories with evidence of genetic interactions between duplicate gene copies using the functional landscape of *S. cerevisiae* (Costanzo *et al.* 2010). (A) The percentage of pairs with interacting gene copies was very high in the category of down-regulated duplicates (red bars), very low in the category of up-regulated duplicates (blue bars), and intermediate in the other three categories under all the stress conditions examined in this study. The mean percentage of duplicates with interacting copies across the stress conditions for each transcriptional category is presented in the inset box. The category “Down” presented a larger mean percentage of duplicates whose gene copies are functionally dependent upon one another than any of the other categories. Significant differences are indicated with ** and *** when the probabilities are *P* < 0.01, *P* < 0.001, and *P* < 10^−4^, respectively. (B) The average proportion of duplicates with interacting gene copies was compared to the proportion of genetic interactions for sets of randomly sampled pairs of singletons with altered transcription profiles under stress. Each transcriptional profile for duplicates was compared to an equivalent set of random pairs of singletons with similar transcriptional profiles. For example, up-regulated duplicates were compared to random pairs of up-regulated singletons under stress.

In all stress conditions the category “Down” showed the highest enrichment for those duplicates with interacting gene copies (Figure 3A). On average over all stress conditions, the genetically interacting duplicates enrichment followed the same pattern, with a distribution among the categories in the following decreasing manner: the category “Down,” followed by the category “Not-altered,” then the category “Only-one,” then the category “Discordant,” and finally the category “Up” (Figure 3A, inset box and Table 3).

**Table 3:** Number of duplicates with genetically interacting gene copies for each transcriptional category of duplicates

Stress	Number of Pairs (Total) Down-Regulated	Number of Pairs (Total) Up-Regulated	Number of Pairs (Total) Discordant	Number of Pairs (Total) Only-One	Number of Pairs (Total) Not-Altered
Ethanol	27 (49)	6 (57)	8 (55)	61 (301)	67 (249)
Glycerol	38 (85)	3 (77)	15 (77)	54 (281)	59 (190)
Lactate	36 (81)	3 (54)	10 (56)	65 (313)	55 (207)
Oxidative + dextrose	36 (64)	5 (67)	12 (71)	72 (319)	44 (189)

The strong genetic interaction between the gene copies could be due to either each gene copy having a large fitness effect such that deleting both magnifies such an effect, or each gene copy having very low fitness effects due to genetic redundancy but deleting both significantly magnifies this effect (*i.e*., functional compensation of a gene deletion or both gene copies are needed to perform the function because they have subfunctionalized). The category “Up” is the one with the lowest number of gene copy interactions, therefore is likely to contain very little genetic compensation, perhaps because gene copies have diverged in their function from the ancestral preduplication gene, and as such the multiplicative effect of deleting single gene copies may be as important in their contribution to fitness as the double gene deletions. The category “Discordant” shows higher levels of genetic interactions between gene copies than the category “Up,” but lower levels than “Down.” Since all discordant duplicates exhibit synergistic epistasis, this suggests certain functional redundancy under normal conditions for transcriptionally discordant duplicates, which also applies to the categories of “One-altered” (average percentage of synergistic epistasis among all stresses: 89.9%; binomial test: *P* < 3.61 × 10^−7^) and “Only-one” duplicates.

To determine whether duplicate gene copies are more dependent upon each other’s functions than expected, we built sets of singleton genes for each of the duplicate sets according to their transcriptional profiles. Each of the singleton transcriptional categories was built taking random pairs of singleton genes. For example, for the “Up” category, both of the singleton genes were sampled from the set of up-regulated singleton genes under stress. We built sets of 1000 pairs and compared each of the duplicate transcriptional categories with the corresponding singleton transcriptional categories. Results show that all the categories, with the exception of the one including duplicates with both gene copies up-regulated, exhibit a significant proportion of their duplicates with interacting gene copies when compared to singletons of the same transcriptional category (Figure 3B). Therefore, up-regulated duplicates seem to exhibit evidence of independent evolution of their gene copies likely due to the finding of novel functions by each copy under stress.

### Functional divergence and genetic redundancy of duplicated genes

The differences in the functional dependencies between gene copies found in the duplicate transcriptional categories hint at a different mode of evolution by gene duplication for these categories. We hypothesize, based on the patterns of genetic interactions, that the functional fate of duplicates in terms of neo- or subfunctionalization is dependent on the transcriptional category they belong to and the genetic redundancy between gene copies. Genetic redundancy has been shown to correlate with evolvability because it provides mutational robustness, which in turn increases the evolvability of genes (Draghi *et al.* 2010; Wagner 2000b, 2005).

To test whether a given transcriptional category of duplicates is more likely to have evolved neo- or subfunctionalization, we examined two parameters linked to genetic interactions: (a) the number of shared interactions between the gene copies, (b) the number of total interactions of the gene copies. Neo-functionalized duplicates involve those in which one of the gene copies has lost all ancestral functions and acquired new functions, hence is likely to have a reduced number of genetic interactions. Conversely, subfunctionalization should affect duplicates with many functions in which each copy has become specialized in a set of ancestral functions while sharing common functions with its sister gene copy, hence is likely to be overrepresented among highly interacting duplicates. Neo-functionalization should also lead to lower levels of sharing of genetic interactions between the gene copies as one of the copies has acquired novel functions that are perhaps independent from subfunctionalization. In agreement with our hypotheses, duplicates from the down-regulated category exhibited a greater number of genetic interactions (mean ± SE: 393.39 ± 14.17) than those of the up-regulated category (mean ± SE: 313.95 ± 13.95; *t*-test: *t* = 3.99, d.f. = 551.94, *P* = 7.36 × 10^−5^). The index of shared interactions was calculated as: $Sh_{A,B}=\frac{1}{2}\left(\frac{Sh}{N_{A}}+\frac{Sh}{N_{B}}\right)$, with *Sh_A,B_* referring to the mean number of shared interactions between gene copies A and B, *Sh* referring to the number of shared interactions, and *N* being the total number of interactions. Duplicates from the down-regulated category shared more interactions (mean ± SE: 0.15 ± 0.005) than those of the up-regulated category (mean ± SE: 0.12 ± 0.004; *t*-test: *t* = 3.29, d.f. = 209.27, *P* = 1.1 × 10^−3^). These results indicate that while up-regulated duplicates may have neo-functionalized, down-regulated duplicates have likely subfunctionalized.

### Sequence divergence levels of duplicates correlate with their transcriptional profiles

**Figure 4:**
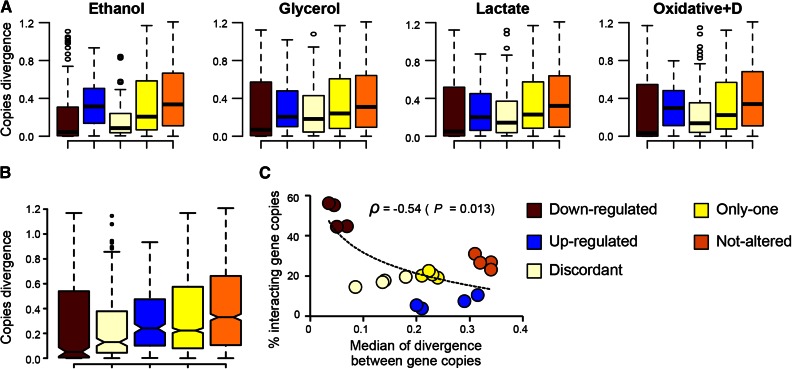
Functional divergence analysis of duplicates with different patterns of transcriptional plasticity. (A) Poisson-corrected amino acid distance between gene copies of duplicates for each of the transcriptional plasticity profiles (“Up,” “Down,” “Discordant,” “Only one,” and “Not-altered”). (B) Comparison of the divergence levels between gene copies for the different transcriptional profiles. (C) Correlation analysis between the percentage of of duplicates with interacting gene copies and the divergence levels between the copies (red, blue, light yellow, yellow, and orange circles refer to the duplicate class of “Down,” “Up,” “Discordant,” “One-altered,” and “Not-altered,” respectively).

To determine whether the transcriptional duplicate categories included specific functional divergence profiles between gene copies, we inferred the amino acid distances between duplicate gene copies for all transcriptional categories and stress conditions. Divergence between duplicate gene copies was calculated using Poisson-corrected distances. Under all four stresses, the duplicates of category “Down” presented the lowest distance between gene copies (Figure 4A), followed by the category “Discordant,” then the category “Only-one,” then the category “Up,” and finally the category “Not-altered” (Figure 4A). Taking all stresses together, we found three groups of transcriptional categories according to the divergence values between gene copies of duplicates (Figure 4B). The first category is “Down”: this category exhibited the lowest divergence levels between gene copies which was significantly smaller than the following group that included “Discordant” category duplicates (median divergence values for “Down”: 0.05, median for “Discordant”: 0.12, Wilcoxon rank test: *P* < 2.2 × 10^−16^). The following group included duplicates belonging to the transcriptional categories of “Only-one” (median divergence between gene copies: 0.22), “Up” (median divergence between gene copies: 0.24), and “Not-altered” (median divergence of gene copies: 0.33). The category “Discordant” exhibited significantly lower divergence between gene copies than the categories “Up,” “Only-one,” and “Not-altered” (Wilcoxon rank test: *P* < 2.2 × 10^−16^).

Importantly, the mean sequence divergence levels between the duplicate gene copies correlated negatively with the mean percentage of duplicates in which both gene copies interacted genetically (Pearson correlation: *ρ* = −0.54, *P* = 0.013; Figure 4C), indicating that the larger the divergence between the gene copies the lower is their functional dependency. This correlation became more significant when taking only those duplicates for which at least one gene copy has showed changing transcriptional patterns under stress (Pearson correlation: *ρ* = −0.78, *P* = 3.4 × 10^−4^). This result is in agreement with a previous study in which the differences between pairs of WGDs in which both gene copies interacted genetically and those in which gene copies did not was analysed (Musso *et al.* 2008).

These results strongly suggest that gene copies that become up-regulated (category “Up”) under stress have undergone accelerated evolution and divergence from their ancestral, preduplication functions perhaps allowing the adaptation to stress conditions that are often encountered by the cell in nature.

### The origin of specific and general adaptations in S. cerevisiae

**Figure 5:**
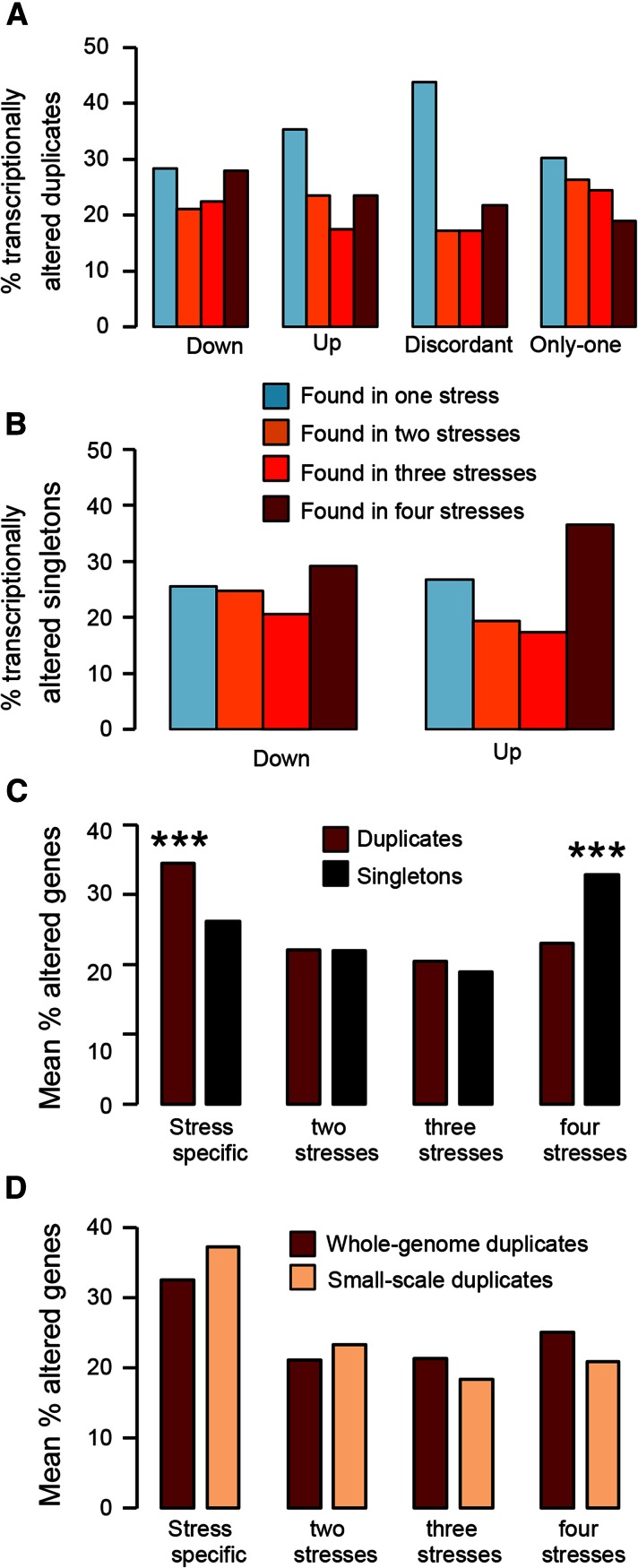
Transcriptional plasticity of duplicates is stress-specific. We analyzed the distribution of transcriptionally altered duplicates in the different stress conditions. (A) Proportion of the transcriptionally altered duplicates from each of the duplicates classes (“Up,” “Down,” “Discordant,” and “Only one”) that are altered in one stress only, two stresses, three stresses, or in all four stresses tested in this study. (B) Proportion of singletons that are up- or down-regulated that respond to specifically one stress only, two stresses, three stresses, or all four stresses. (C) The mean percentage of altered genes across the different transcriptional classes in duplicates and singletons that are altered under one or more type of stress. (D) The mean percentage of whole-genome duplicates and small-scale duplicates that are transcriptionally altered when *S. cerevisiae* is faced with one or more stresses. *** indicates *P* < 0.001 under a Fisher’s exact test.

To determine whether the transcriptional plasticity of duplicates is the result of an adaptive process to face environmental perturbations, we sought to investigate whether this plasticity is stress specific (*i.e.*, the result of adaptive processes) or a general response to stress. We examined common transcriptionally altered duplicates for each of the transcriptional categories among stress conditions. We found that a substantial proportion of duplicates showed stress-specific transcriptional plasticity (Figure 5A). This pattern was the inverse in the case of transcriptionally altered singletons, with many common such singletons responding to all four stress conditions (Figure 5B). Comparison of the proportion of duplicates in each of the categories for stress response (*i.e.*, stress specific, common genes response to two, three, or four stresses) revealed more significant stress-specific transcriptional alterations in duplicates than in singletons (a mean of 34.4% of duplicates with transcriptional flexibility were stress specific compared to 26% of singletons, Fisher’s exact test: odds ratio *F* = 1.47, *P* = 3.3 × 10^−4^), while singletons showed more common responses to all stresses than duplicates (a mean of 32.8% of singletons responded to all stress conditions compared to 23% of duplicates, Fisher’s exact test: odds ratio *F* = 1.64, *P* = 1.1 × 10^−5^) (Figure 5C). These results reveal a fundamental difference in the transcriptional plasticity of duplicates and singletons, with evidence for the role of natural selection in duplicates’ transcriptional differences as an adaptive mechanism.

Because of the fundamental difference in transcriptional plasticities between WGDs and SSDs (Figure 1B), we split the dataset for duplicates into these two groups and conducted the same comparison as above. Both the WGDs and SSDs showed very similar transcriptional flexibility patterns to the entire dataset: WGDs and SSDs had their largest transcriptional plasticity in genes that responded in a stress-specific manner (Figure 5D).

**Figure 6:**
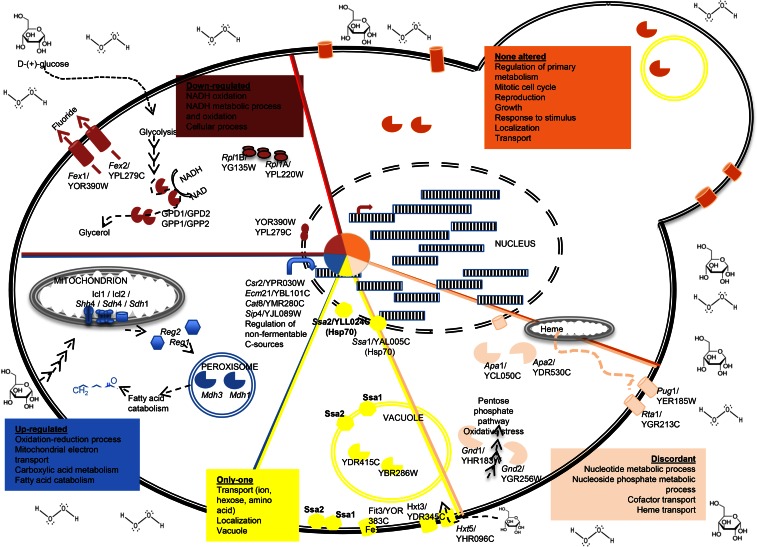
Schematic representation of main GO processes and selected duplicate gene pairs affected by oxidative stress in the presence of dextrose. The schematic *S. cerevisiae* cell has been partitioned in a pie shape with the proportions being in accordance to the distribution of duplicates in categories (“Up,” “Down,” “Discordant,” “One-altered,” and “Not-altered”). Color codes follow the same categorization. For those categories, “One-altered” and “Discordant,” on which each gene in the pair show a differential fold-change, it has been shadowed with green if not differentially expressed, with blue if down-regulated, or with red if up-regulated. Some of the pathways affected are indicated with the corresponding genes names and ID tag, except for the most common gene names, or when not available a common name and the ID tag is provided. Curved arrows in the nucleus represent some transcription factors affected; color indicates the corresponding category. Subcellular localization of proteins has been retrieved from Saccharomyces Genome Database following Gene Ontology term analysis.

To understand the relationship between adaptation to stress and transcriptional plasticity, we analyzed how the different transcriptional alterations in duplicates may have an important role in the adaptation to oxidative stress supplemented with dextrose (Figure 6). Oxidation generates reactive oxidative molecules or species (ROS) including peroxide, superoxide, hydroxyl radicals, and single oxygen in the cell. Increasing ROS in the cell can lead to important structural damage. We found a number of important duplicates that are involved in mitochondrial respiration (*Icl1*/*Icl2*, *Shh4*/*Shh*1, and *Sdh4*/*Sdh1* duplicated genes, among others) as well as duplicated genes encoding transcriptional gluconeogenesis activators (*Cat8*/*Sip4* and *Csr2*/*Ecm21*) to be up-regulated under oxidative stress, perhaps to reduce the generation of ROS. Moreover, duplicated genes involved in NADH metabolism and oxidative processes in the glycolysis pathway (*Gpd1*/*Gpd2*, *Gpp1*/*Gpp2*) are down-regulated under stress (Figure 6). Interestingly, as previously noticed (Belli *et al.* 2004), the gene copies (*Pug1*/*Rta1*) involved in heme transport and iron ion homeostasis showed discordant expression patterns, while the gene copies (*Fit3*/*Fit1*) involved in ion transport showed transcriptional alterations only for *Fit3* (Figure 6).

The transcriptional plasticity of the duplicates belonging to the category “Only-one” was very noticeable under oxidative stress affecting functional classes required to minimize ROS, including many duplicated genes involved in ion transport (*Fit1*/*Fit3*), hexose transport (*Hxt3*/*Hxt5*), and heat stress response (*Ssa2*/*Ssa1*). Similarly, the transcriptional category “Discordant” also showed a prominent response pattern to oxidative stress, including duplicates involved in nucleotide/nucleoside metabolism (*Gnd1*/*Gnd2*, *Apa1*/*Apa2*), and in heme transport and iron ion homeostasis (*Pug1*/*Rta1*). Most of these duplicates have important roles in DNA replication and stress.

Interestingly, some of these genes are involved in many stresses but their transcriptional plasticity exhibits different patterns under different stresses. For example, the duplicates (*Gpd1*/*Gpd2* and *Gpp1*/*Gpp2*) that drive glycerol production using dextrose through the glycolysis pathway (NADH metabolism and oxidative processes) behave transcriptionally different under the different stresses. Both of the gene copies of these duplicates are down-regulated under oxidative stress supplemented with dextrose, while only one gene copy is down-regulated when the cell is subjected to stress by lactate or glycerol, and none of the gene copies showed any differential expression level with the wild type when the cell grew under ethanol stress. Similarly, the gene copies of the duplicates (*Shh4*/*Sdh4* and *Sdh1/Sdh1b*) involved in oxidation of succinate and electron transfer to ubiquinone are up-regulated under oxidative stress. However, under ethanol stress, only gene copies *Shh4* and *Sdh4* are up-regulated while their corresponding paralogs *Shh1* and *Sdh1* show wild-type expression levels (Figure 6).

## Discussion

In this study, we demonstrate that ancient duplicates of *S. cerevisiae* exhibit a large transcriptional plasticity when subjected to stress. This transcriptional plasticity may be the result of preadaptations to environmental stress. Such preadaptations may have been generated through an increase in the polymorphism of the regulatory sequence regions of duplicated genes, perhaps the same sequence changes that have led to the regulatory divergence between the gene copies of duplicates. The transcriptional divergence between gene copies has been studied in plants (Blanc and Wolfe 2004; Ha *et al.* 2007, 2009; Wang *et al.* 2012) and animals (Huminiecki and Wolfe 2004). In *S. cerevisiae*, while the genome-wide transcriptional plasticity has been reported under stress (Causton *et al.* 2001; Cormier *et al.* 2010; Ferea *et al.* 1999; Ideker *et al.* 2001; Landry *et al.* 2006; Stern *et al.* 2007), the differential patterns in this plasticity between duplicates and singletons have received little attention. Findings from these studies led authors to conclude that the transcriptional plasticity of the genes in *S. cerevisiae* is the result of a general response to a wide range of stresses, sparking the possibility that this plasticity is an emerging property resulting from a universal feature of the underlying regulatory network. In this study we show that: (i) ancient duplicates of *S. cerevisiae* exhibit a large transcriptional plasticity when subjected to stress; and (ii) the transcriptional plasticity of duplicated genes differs from that of singletons, is more complex than thought before, and is likely the result of selection for an adaptive response to specific environmental challenges.

The transcriptional changes affecting one or both of the gene copies resulting from gene duplication may be selectively advantageous in unicellular organisms because the absence of tissue-specific transcriptional subfunctionalization precludes a relief of the genetic redundancy of duplicated genes. Therefore, in unicellular eukaryotes, such as *S. cerevisiae*, the efficiency of purifying selection or positive selection must be a strong force driving the fate of duplicated genes. In agreement with this prediction, the genetic redundancy generated in *S. cerevisiae* after the WGD event that took place roughly 100 MYA was erased by purifying selection, as 92% of duplicated genes returned to single copy genes (Wolfe and Shields 1997). Despite this, the number of duplicates in *S. cerevisiae* (roughly 30% of all the genes) is higher than predicted by theory, raising the possibility that most retained genes have become functionally specialized, hence less redundant, shortly after duplication (Force *et al.* 1999; Lynch and Katju 2004; Barkman and Zhang 2009; Des Marais and Rausher 2008; He and Zhang 2005; Conant and Wolfe 2006), transcriptionally divergent (Blanc and Wolfe 2004; Ha *et al.* 2007, 2009; Wang *et al.* 2012; Francino 2005), preserved due to their higher mutational robustness (Fares 2015; Fares *et al.* 2013; Keane *et al.* 2014; Wagner 2000b, 2005), maintained owing to a selective advantage for higher gene dosage (Conant and Wolfe 2008), or kept to preserve stoichiometric balances in duplicates encoding protein complexes (Gibson and Spring 1998; Veitia 2003a,b).

In this study, the transcriptional plasticity identified in *S. cerevisiae* is likely the result of population polymorphism at the regulatory regions of duplicates (Figure 3C), which were selectively relaxed after gene duplication. This polymorphism has likely given rise to preadaptations to environments never before faced by the yeast and became fixed in the populations after facing such environmental perturbations. It is therefore likely that duplicates that show transcriptional plasticity, in particular those that become up-regulated under stress, are usually performing important functions in the cell and are hence maintained by purifying selection. However, under stress, such genes may encode new functions that provide the yeast with the ability to survive stress, a property encapsulated within the term exaptation (Gould and Vrba 1982). The question that remains is: how important an adaptive force is the transcriptional divergence against the functional divergence of duplicates in *S. cerevisiae*?

Functional divergence after gene and genome duplication has been the subject of intense scrutiny and a number of examples unequivocally correlate the origin of important gene families and functional specialization with the divergence between gene paralogs. Indeed, key globin proteins that specialized in different aspects of oxygen metabolism have originated through WGD events (Hoffmann *et al.* 2011, 2012a,b; Storz *et al.* 2011, 2013). Functional divergence has also been observed in a number of studies and has been correlated with an asymmetric increase in the rates of sequence evolution in the duplicate gene copies (Blanc and Wolfe 2004), in good agreement with the fundamental tenet of the molecular evolution theory (Gu *et al.* 2002; Dermitzakis and Clark 2001). Expression divergence, but not transcriptional plasticity, between the copies of duplicated genes has also been demonstrated in a number of organisms.

We propose the hypothesis supporting a link between expression and functional divergence – that is, one level of divergence necessarily drives the other level. Indeed, gene expression levels largely determine the rates of evolution of the proteins they encode (Drummond *et al.* 2006; Drummond and Wilke 2008; Pal *et al.* 2001; Rocha and Danchin 2004; Wilke and Drummond 2006). The theoretical justification for this link between gene expression and its rate of evolution can be found in the misfolding–mistranslation hypothesis, according to which highly expressed genes evolve slower constrained by the need to maintain low levels of misfolded or mistranslated proteins bearing destabilizing mutations (Drummond *et al.* 2005). On the other hand, functional divergence, or acquisition of novel functions, may involve a fine-tuning of the expression of the encoding gene to perform the required function at the right rate. Whether expression divergence came before functional divergence or vice versa remains to be investigated but our data suggest a link between these two levels of divergence because different transcriptional categories exhibit different patterns of sequence evolution and divergence between gene copies (Figure 4C). We hypothesize that genetic redundancy has allowed transcriptional divergence between gene copies due to relaxed selective constraints. This has allowed divergence at the coding level driven by changes in gene expression, as gene expression is a strong determinant of sequence evolution (Drummond *et al.* 2005). Such functional divergence may have led to the acquisition of functions that enabled the adaptation to stress conditions (Figure 5C).

In this study, we determine the plasticity that each of the gene copies has at the regulatory level, the link of this plasticity with the functional dependencies among gene copies, and the role of such a link in the response to stress. Our study reveals different modes of evolution for the different transcriptional categories. Most responsive duplicates to stress present only one copy altered, following the classic view of evolution by gene duplication. The genetic dependencies and low sequence divergence between gene copies for these duplicates also reveal the mode of evolution and innovation: these duplicates exhibit the highest proportion of cases with synergistic epistasis between gene copies, which summed to the low sequence divergence between the gene copies indicates higher genetic redundancy (VanderSluis *et al.* 2010). This nontrivial pattern of evolution of novel functions is in agreement with previous predictions, according to which higher genetic redundancy allows the functional compensation between gene copies, the neutral exploration of genotypic space, and eventual finding of additional novel functions (Fares 2015; Fares *et al.* 2013; Keane *et al.* 2014; Wagner 2005). The category of up-regulated duplicates exhibits evidence of neo-functionalization based on the rapid evolution of the gene copies when compared to their ancestor and the low functional dependency of each copy on its sister copy, suggesting the acquisition of novel functions. Duplicates with both copies being down-regulated under stress present low divergence between the gene copies and significant functional dependencies among the gene copies, suggesting the subfunctionalization of the gene copies through the partition of ancestral functions. The partition of ancestral functions in these duplicates is not complete, as the gene copies share more functions than expected. This greater sharing may reflect a selective advantage for gene dosage, particularly in the ancestral state immediately postdating genome duplication (Ihmels *et al.* 2007; VanderSluis *et al.* 2010). Finally, the category in which gene copies exhibit discordant transcriptional alterations under stress (“Discordant”) presents a low number of genetic interactions between gene copies and low sequence divergence of the gene copies when compared to a preduplication ancestral gene, suggesting that both of the gene copies may be performing very similar functions under different conditions (Force *et al.* 1999). The category of discordant duplicates may include cases in which copies have diverged in their regulation such that one copy is active under stress and its sister copy is active under normal conditions, thereby avoiding the costly evolutionary optimization of the encoded function under two different conditions (Conant and Wolfe 2006). Remarkably, duplicates with no evidence for expression alteration under stress conditions exhibit a greater number of interactions between gene copies than expected. Because these duplicates are not affected by environmental perturbations, their enrichment for genetic interactions supports genetic buffering between the gene copies that is independent of the environment, a phenomenon important for genetic robustness (Dean *et al.* 2008; DeLuna *et al.* 2008; Fares 2015; Keane *et al.* 2014; VanderSluis *et al.* 2010).

Our results point to the expression divergence between the gene copies of duplicated genes as a strategy to yield adaptive responses to stress conditions. This divergence is the result of differential accumulation of mutations (polymorphism) in the promoters of the duplicate gene copies, such that one copy exhibits high expression polymorphism under normal environmental conditions (Keane *et al.* 2014). Since yeast generally undergo stress because of the changes in the osmotic characteristics of the medium resulting from the metabolic byproducts generated, such stress conditions provide selective advantage to those strains with polymorphic expression divergence of duplicated genes that allow response to stress. A number of findings disregard expression noise as an alternative hypothesis to the adaptive value of the observed transcriptional alterations in duplicates. First, the profiles indicating adaptations to stress (*i.e.*, the transcriptional categories including duplicates with at least one gene up-regulated) include duplicated genes that under normal conditions show as much contribution to fitness as those with no response to stress (Figure 1, C–F). Second, under stress conditions, the contribution of up-regulated duplicates to fitness is significantly higher than their contribution to fitness under normal conditions. Finally, duplicates with altered expression profiles are mostly stress-specific and affect functions that are strictly related to the interaction with the environment and signal transduction.

In conclusion, we reveal the underlying transcriptional plasticity of duplicates in *S. cerevisiae* and the potential of this plasticity to give origin to adaptations (by means of specialization of duplicates) to environmental perturbations. This study sets a new research endeavor mainly aiming at finding the as yet unexplored metabolic capabilities resulting from the evolution of duplicated genes.

## Supplementary Material

Supplemental material is available online at www.g3journal.org/lookup/suppl/doi:10.1534/g3.116.035329/-/DC1.
