# Supplementary material for: The Phenotypic Plasticity of Duplicated Genes in Saccharomyces cerevisiae and the Origin of Adaptations
Source: G3 (Bethesda). 2016 Oct 31;7(1):63–75. doi: 10.1534/g3.116.035329 (PMC5217124; doi:10.1534/g3.116.035329)
Supplement: Supplementary file 8 [file 63FileS8.pdf]

The Phenotypic Plasticity of Duplicated Genes in *Saccharomyces cerevisiae* and the Origin of Adaptations

Florian Mattenberger[*,1], Beatriz Sabater-Muñoz[*,†,1], Christina Toft[‡,§], Mario A. Fares[*,†,2]

File S8.

Comparison of different methods of differential expression through the analysis of RNA sequence data

We compared three main methods of differential expression by calculating the correlation in the fold change estimated by these methods. The three methods used were edgeR, DESeq, and Cufflinks.

Analysis of the correlation in the logarithm of fold change between edgeR and DESeq supports that the data are very robust to the use of these two methods, with a spearman´s correlation of 0.995, $P < 2.2 \times 10^{-16}$ (Fig 1.).

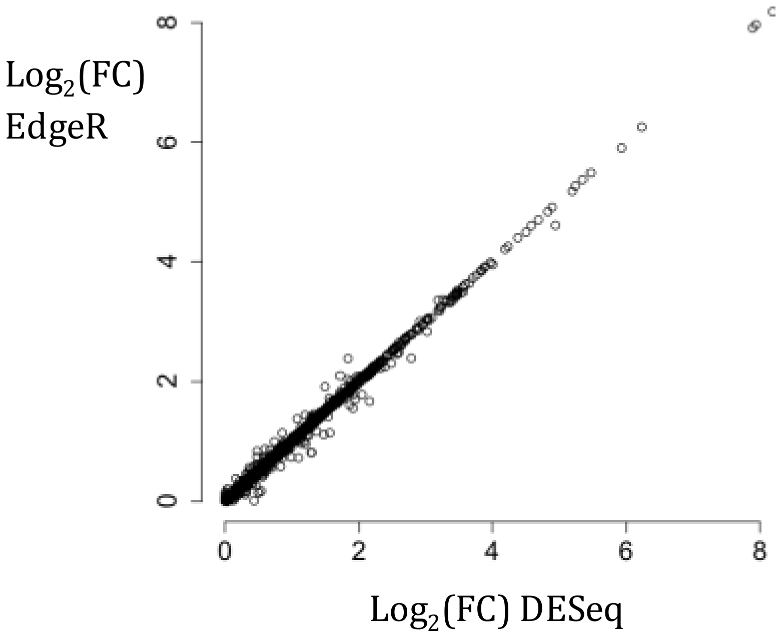

Fig. 1. Correlation analysis in the fold change expression using a reference transcriptome from strain S288c between two methods edgeR and DESeq. All numbers have been log-transformed for the comparison.
